# Psychotherapists’ perspectives on loss of sense of control

**DOI:** 10.1002/brb3.3368

**Published:** 2024-01-06

**Authors:** Eve Riachi, Juha Holma, Aarno Laitila

**Affiliations:** ^1^ Department of Psychology University of Jyväskylä Jyväskylä Finland

**Keywords:** loss of sense of control, psychological disorders, psychotherapy, qualitative research

## Abstract

**Introduction:**

Sense of control is an integral part of well‐being. Studies have reported on the connection between loss of control and psychological symptoms. However, loss of sense of control has not yet been studied from the perspective of psychotherapists.

**Methods:**

This study had three research objectives: to find out how psychotherapists define loss of sense of control, whether they consider loss of sense of control to play a role in the start of psychological symptoms, and, if so, in what cases. Lebanese psychotherapists were interviewed and the data were then analyzed using frame analysis.

**Results:**

The analysis revealed two definitions for loss of sense of control and conflicting views on whether it plays a role in the onset of disorders. Problems within relationships and stress were the most mentioned examples of loss of sense of control.

**Conclusion:**

The findings shed light on psychotherapists’ diverse opinions and explanations regarding the role of loss of sense of control in the development of psychological symptoms.

## 1 INTRODUCTION

Shapiro (1994) defines sense of control as individuals’ beliefs about how much control they possess and their ability to gain control if necessary. Another definition focuses on mastery or agency and describes sense of control as a basic need that is motivated by positive feelings generated by accomplishing one's goals (Lachman et al., 2015). Sense of control has also been defined as the perceived level of control one has over the events in one's life, or the view that one can overcome environmental uncertainty (Ward, 2013; Zhu et al., 2020). In contrast, loss of sense of control is explained as powerlessness and the belief that one is unable to exercise control over one's life or effect change (Ross & Sastry, 1999).

Loss of sense of control has been linked to depression, stress, anxiety, and burnout (Keeton et al., 2008; Precht et al., 2021; Southwick & Southwick, 2018). Studies have also shown feelings of ineffectiveness and fear of losing control to be linked with binge eating disorders as well as obsessive and compulsive symptoms (Froreich et al., 2016). In addition, self‐harm behaviors and suicidal ideation have also been associated with loss of control over oneself and one's life situation (Pavulans et al., 2012; Wand et al., 2018).

Loss of sense of control was also linked to the adoption of dysfunctional coping mechanisms and maladaptive behaviors such as alcohol or drug abuse (Brailovskaia & Margraf, 2021). In one study, sense of control acted as a mediator between physical activity and psychological symptoms such as depression, anxiety, or stress (Precht et al., 2021). Unpredictable childhood experiences were shown to affect belief systems and correlate with anxiety and depression (Ross et al., 2016). Other studies discuss intolerance of uncertainty, and how this could be associated with maladaptive emotional regulation and psychological symptoms (McEvoy & Mahoney, 2012; Sahib et al., 2023). Intolerance of uncertainty is a characteristic linked to pathological worry and develops as a result of negative beliefs which can then result in a negative emotional, cognitive, or behavioral reaction to uncertainty (Boswell et al., 2013; Buhr & Dugas, 2009).

While the relation between loss of sense of control and psychological disorders has been demonstrated, its role in the onset of psychological disorders has not yet been examined. Skovholt (2012) explains how many factors contribute to a therapists’ professional expertise such as psychotherapeutic orientation and professional experience while working with clients. Based on their theoretical and practical knowledge, psychotherapists may be able to contribute on the topic of loss of sense of control. How therapists define loss of sense of control will not only add further insight but will also create a basis for treatment.

This study investigated the role of loss of sense of control from the perspective of Lebanese psychotherapists. The ongoing geopolitical conflict and economic crisis affects citizens' mental and physical health. Lebanon is currently classified by the World Bank as a lower‐middle income country (The World Bank, 2022).

Therapists in this study were first asked to define loss of sense of control in their own words. Second, they were asked to clarify whether they perceive loss of sense of control as playing a role in the onset of psychological disorders. Finally, they were asked to provide examples of disorders in which loss of sense of control plays an initiating role.

## 2 METHODS

### 2.1 Participants and procedures

The participants in this study were Lebanese psychotherapists. Inclusion criteria included geographical location, experience working as a psychotherapist and English language skills. Of the sixty therapists contacted, sixteen agreed to participate. All were located in Beirut and had differing therapeutic and educational backgrounds. Some were employed in psychotherapy centers or psychiatric units and others had their own psychotherapy clinics.

All participants were clearly informed about the purpose of the study, that is, the development of psychological disorders and the possible role in this of loss of sense of control. All were also informed that their participation was voluntary, and the interviews would be voice‐recorded and conducted in English. They were also notified that the results of the study would be published and their personal information and rights to privacy would be protected. Prior to the interviews, participants were asked to give their verbal consent and sign a written consent form.

The interview was semistructured and contained thirteen questions. The three questions which focused on the role of loss of sense of control constitute the data for this study. Therapists were asked how they would define loss of sense of control, then whether or not they believe it to play a part in the development of psychological disorders. Finally, they were asked to describe any cases in which loss of sense of control triggered the onset of a psychological disorder. The data were collected in English to minimize translation bias as much as possible and, more importantly, to utilize participants’ own words and phrases in the data analysis.

The interviews were voice‐recorded except for one, in which the participant preferred not to be recorded and therefore the interview answers were written directly at the time of the interview. All the interviews were collected in English except for one which was mostly in Arabic and then translated into English. Some participants switched to Arabic when providing additional explanations and these statements were also translated into English.

### 2.2 Analysis

The data from each of the three interview questions were analyzed separately using Atlas.ti software. Frame analysis is a research methodology developed by Erving Goffman (Goffman, 1974) and is a form of discourse analysis which allows the investigation of diverse perspectives in relation to a certain topic. The theoretical framework of this study is based on the idea that knowledge is constructed through reflection and combining experiences with preexisting information (Patel, 2015). Coding was based on the therapists’ choice of words and phrases to limit interpretation bias. Similar codes displaying a similar opinion were combined to create the frames.

One interview was excluded from the analysis of the second research question and three interviews were excluded from the analysis of the third research question since the participants’ answers did not provide specific information on the topic or examples of the role of loss of sense of control.

## 3 RESULTS

### 3.1 Defining loss of sense of control

To address the first research question, the psychotherapists were asked to define of loss of sense of control. The data analysis yielded two frames, which were labeled helplessness and disinhibition. The helplessness frame was mentioned 11 times by the therapists while the disinhibition frame was mentioned 5 times.

#### 3.1.1 Helplessness frame

The psychotherapists who defined sense of loss of control as helplessness spoke about feelings of helplessness or powerlessness, using expressions such as feeling passive and hopeless. Some of the psychotherapists described these feelings in connection with certain situations such as the accumulation of adverse events, uncertainty, or unpredictable environments. Others referred to symptoms, belief patterns, and psychopathology as the main indicators of loss of sense of control.

*Interview 8: An individual feeling that they are not able to control the events that are taking place in their lives. It's hopelessness or learned helplessness*.

*Interview 6: Psychopathology is a loss of sense of control, because you don't know how to handle your life anymore*.

#### 3.1.2 Disinhibition frame

The psychotherapists who defined loss of sense of control as disinhibition spoke about the inability to control one's psyche, emotions, or behaviors. They gave examples such as addictions, self‐harm, impulse control, binge eating, abusing one's partner or parents abusing their children.

*Interview 3: How I understand loss of sense of control is in terms of impulse control disorders, so when you decide to do something and you cannot stick to what you have decided. For example, binge eating or alcohol abuse. Loss of control could be emotional imbalance, or it could be emotional dysregulation for example self‐harm*.

*Interview 16: When you are overwhelmed and you cannot control your emotions or your actions*.

### 3.2 The role of loss of sense of control

The psychotherapists were asked whether they believe loss of sense of control plays a role in the onset of disorders. The data analysis yielded two frames, one was mentioned 10 times indicating agreement and the other mentioned 7 times in disagreement. Some of the psychotherapists provided statements in both frames.

#### 3.2.1 Loss of sense of control plays a role

The psychotherapists who expressed belief that loss of sense of control plays a role in the onset of psychological disorders discussed several reasons for this. Some stated that whether or not an individual develops a psychological disorder depends on the events an individual has been exposed to, how unpredictable these events were and how they were processed. They also focused on the feeling of being unable to change certain issues or events in one's life and how the intensification of loss of sense of control could lead to the development of a disorder. A few therapists discussed the role of fear, namely, in the context of posttraumatic stress disorder, and explained that a certain event may induce both loss of sense of control and fear, which could lead to a vicious cycle of fear and avoidance. Some mentioned how loss of sense of control could create patterns of beliefs such as, “I am not capable, I am not strong enough, I am not important,” which could lead to emotional dysregulation and possibly a disorder.

Others pointed to emotional dysregulation as the initiating factor in this process; they argued that emotional dysregulation leads to impulsive behavior, which in turn generates feelings of loss of control. Others emphasized feelings of helplessness or powerlessness in addition to feeling unsafe and insecure. These feelings lead to fear that hinders one's ability to take the necessary steps in creating the life they wish to have. Finally, a few discussed the vulnerability stress model, in which stressors generate loss of sense of control and trigger psychological disorders in individuals who have a predisposition for developing a given disorder.

*Interview 2: PTSD being a prime example for that. Something happens and you lose your sense of control and it creates fear, then that fear creates avoidance and the avoidance creates more fear and it becomes a cycle*.

*Interview 16: It plays a part, it depends on the outcome of the loss of sense of control; what happened when I lost control, how did I react or behave, how did I process it, how did it affect me; so all those factors play a role in whether or not I would develop a disorder*.

#### 3.2.2 Loss of sense of control plays no role

In contrast to the therapists in the previous frame, therapists stated that loss of sense of control does not play a role in the onset of disorders. Some stated that environmental factors and genetics play a major role. Others viewed loss of sense of control as a symptom or a consequence of pathology and not the other way around. They explained that symptoms result in feelings of loss of sense of control.

*Interview 3: I think loss of sense of control is a consequence of a disorder. When people have a psychological disorder they have this feeling of not being able to manage their life properly, so loss of sense of control can be an impairment that is present due to the disorder itself*.

*Interview 11: Loss of sense of control does not play a role in all psychological disorders. I think this is a generalization. For example: some mood disorders, bipolar disorder, schizophrenia, schizoaffective disorder. A strong genetic component plays a part in these disorders*.

### 3.3 Cases of loss of sense of control

To address the third research question, the therapists were asked to describe cases where, in their opinion, loss of sense of control triggered the onset of a psychological disorder. The examples provided were labeled relational issues, stress, life events, abuse, failure, trauma, and drug abuse. The frames and their frequencies are presented in Table 1.

**TABLE 1:** Frames describing examples of loss of sense of control.

Frames	Frequencies
Relational issues	8
Stress	6
Life events	5
Abuse	4
Failure	3
Trauma	3
Drug abuse	2

#### 3.3.1 Relational issues frame

In the cases provided by the therapists in this frame, loss of sense of control is linked to relational issues. Therapists discussed interpersonal problems stemming from, for example, personal or family relationships, including breakups, divorce, cheating, family problems, sickness in the family, mental illness in the family, overprotective parents, parents with depression, and unsupportive parents. Relational issues were the most commonly mentioned examples of loss of sense of control.

#### 3.3.2 Stress frame

This frame contained the second most frequently mentioned examples of loss of sense of control. Some of the therapists spoke about the accumulation of stress and the inability to manage, leading to burnout as well as work‐related stress. Other examples included everyday stress, living in an unstable country, and new situations that induce immense stress, such as giving birth for the first time or getting married and not expecting the big life changes that accompany these events.

#### 3.3.3 Life events frame

The life events exemplified in this frame were illness, death, accidents, and being a victim of crime. These examples focus mainly on events that are independent of a relational perspective. The therapists explained how these events could result in an individual feeling unsafe and unable to predict the future.

#### 3.3.4 Abuse frame

This frame of loss of sense of control contained statements in which the therapists mentioned the word abuse, mainly experienced in childhood or from an abusive partner. The therapists’ main focus was on abuse, irrespective of its specific context.

#### 3.3.5 Failure frame

The examples in this frame referred to failure achieving goals, such as in school and university or losing one's job. The therapists for example elaborated on the situation of a person receiving low grades and not knowing the reasons for this or not knowing how to deal with or improve the situation.

#### 3.3.6 Trauma frame

Some therapists saw trauma as a trigger for both loss of sense of control and the onset of psychological symptoms and disorders. Some stated that trauma is the main trigger and that loss of sense of control is one of the symptoms induced by a trauma; this loss of sense of control then leads to secondary symptoms such as psychopathology. Others also cited cases where loss of sense of control led to symptoms.

#### 3.3.7 Drug abuse frame

In this frame, psychotherapist discussed substance abuse as an example of loss of sense of control. They described the effects of certain psychoactive drugs on the biochemistry of the brain and explained that mind‐altering substances can activate anxiety and feelings of loss of sense of control that can in turn develop into symptoms.

## 4 DISCUSSION

The findings of this study provide an insight into how psychotherapists understand loss of sense of control. The study had three main aims: to find out how psychotherapists define loss of sense of control, whether they consider it has a role in the onset of psychological disorders, and if so, in what kinds of cases. First, the therapists’ definitions of loss of sense of control could be categorized into two frames: one of helplessness and one of disinhibition.

Second, the answers to the question of whether or not loss of sense of control plays a role in the onset of psychological disorders was categorized into two frames, one affirmative and the other negative. The affirmative view was slightly more frequently expressed. The therapists offered several reasons for loss of sense of control having a role in the development of psychological symptoms or disorders. Those who disagreed with the idea that loss of sense of control plays a part in the onset of disorders focused on the presence of other factors or stated that loss of sense of control is a symptom rather than a trigger. Lastly, the examples given by therapists of cases of loss of sense of control were categorized into several frames. The most frequently mentioned frame was relational issues.

In this study, the definition of loss of sense of control in the helplessness frame resembled definitions within the literature. That is, the definitions focused on helplessness, powerlessness, and the belief that one does not have control or cannot gain control over one's life. However, the therapists in this study also mentioned loss of control over oneself, one's psyche, emotions, or behaviors.

A link between loss of sense of control and psychological disorders, such as depression, anxiety, insomnia, obsessive compulsive symptoms and eating disorders, has been found in previous studies (Froreich et al., 2016; Keeton et al., 2008; Precht et al., 2021; Southwick & Southwick, 2018). Some of the therapists in this study explained that, as they saw it, loss of sense of control does not play a role in all psychological disorders, giving bipolar, schizoaffective disorder and schizophrenia as examples. They discussed what they viewed as the essential role of genetics in these disorders.

Previous research has reported on the link between loss of sense of control and maladaptive behaviors, while other studies explained the associations between feelings of ineffectiveness, vulnerability and fear of losing control (Brailovskaia & Margraf, 2021; Froreich et al., 2016). However, in this study, therapists provided different explanations for how loss of sense of control influences the development of symptoms. Some discussed the role of loss of sense of control as a trigger for any genetic predisposition that could develop into a psychological disorder. Another explanation focused on the accumulation of unpredictable events that an individual is unable to control, possibly leading to a psychological breaking point. Others discussed loss of sense of control in relation to cognitive belief patterns and emotional dysregulation. Some stated that loss of sense of control creates belief patterns that lead to emotional dysregulation and hence symptoms, while others believed that emotional dysregulation leads to impulsive behaviors which result in loss of sense of control, and only then in the development of symptoms. Finally, some stated that feelings of powerlessness and helplessness create fear, which in turn hinders an individual's ability to change or deal with certain situations, thereby inducing more loss of sense of control and ultimately psychological symptoms.

Previous research associated unpredictable childhood events and intolerance of uncertainty with psychological symptoms and maladaptive behavior (McEvoy & Mahoney, 2012; Ross et al., 2016; Sahib et al., 2023). Therapists in this study supported these findings and added that the way an individual reacts to uncertain events plays a part in whether or not symptoms would develop. This can be associated with intolerance of uncertainty or the inability to deal with these events in a healthy and adaptive manner.

The therapists differing views were also visible in data pertaining to the examples of loss of sense of control. In the trauma frame for instance, some therapists discussed traumatic experiences as an example of loss of sense of control triggering psychological symptoms, while others stated that trauma causes loss of sense of control, which can then lead to secondary symptoms. Loss of sense of control in this case was not considered as a predecessor but as a symptom of trauma.

### 4.1 Strengths and limitations

This study offered a unique perspective on the role of loss of sense of control in the onset of psychological disorders that has not been discussed in previous studies. Moreover, this research yielded new insights into the opinions of psychotherapists, specifically Lebanese psychotherapists, that are underrepresented in the literature.

The fact that the interviews were conducted in English could be considered a limitation, as English was not the participants’ native language. However, most of them had a good command of the English language and had either studied or worked in English or received training in English. Only one interview was conducted in Arabic and translated into English.

The frames describing examples of loss of sense of control were not mutually exclusive. Some cases of abuse might also have been considered a relational issue; however, the categories were all based on the words and concepts used by the therapists.

## 5 CONCLUSION

This research focused on Lebanese psychotherapists’ views on loss of sense of control. Three research objectives were set; first, to obtain definitions of loss of sense of control; second, to clarify its potential role in the start of psychological symptoms; and third to gather examples of disorders triggered by loss of sense of control. The therapists agreed with the definition of loss of sense of control found in the literature and added the aspect of loss of control over one's thoughts, emotions, and actions. The therapists provided several explanations on the role of loss of sense of control in the onset of disorders. Some discussed unpredictable events as triggering factors for feelings of loss of sense of control and consequently the development of symptoms, while others explained that it is the way individuals deal with and react to uncertain events that may result in loss of sense of control. These differing views were also visible in the examples provided. Trauma was discussed as an example of loss of sense of control by some of the therapists, while others explained that trauma is the trigger for symptoms, and loss of sense of control is a result of an already existing psychopathology.

It would be interesting to examine the role of loss of sense of control in different psychological disorders separately, and to understand the many ways therapists assist their clients in regaining sense of control.

## AUTHOR CONTRIBUTIONS

**Eve Riachi**: conceptualization; data curation; formal analysis; funding acquisition; investigation; methodology; project administration; resources; software; validation; visualization; writing—original draft; writing—review and editing. **Juha Holma**: conceptualization; formal analysis; funding acquisition; methodology; project administration; resources; supervision; validation; visualization; writing—review and editing. **Aarno Laitila**: conceptualization; formal analysis; funding acquisition; methodology; project administration; resources; supervision; validation; visualization; writing—review and editing.

## CONFLICT OF INTEREST STATEMENT

The authors declare that they have no competing interests that are relevant to this article.

### FUNDING INFORMATION

No funding was received for conducting this study.

### CONSENT FOR PUBLICATION

Participants consented to the use of the data for publishing.

### PEER REVIEW

The peer review history for this article is available at https://publons.com/publon/10.1002/brb3.3368.

## Data Availability

Consent for sharing the data was not provided by participants.
